# Gastrointestinal cancers, ACE-2/TMPRSS2 expression and susceptibility to COVID-19

**DOI:** 10.1186/s12935-021-02129-x

**Published:** 2021-08-16

**Authors:** Sepehr Shafiee, Luca Cegolon, Mostafa Khafaei, Nasrin Gholami, Shi Zhao, Nasrin Khalesi, Hamidreza Moosavian, Saeid Fathi, Morteza Izadi, Alireza Ghadian, Mohammad Javanbakht, Amin Javanbakht, Reza Akhavan-Sigari

**Affiliations:** 1 Shahid Beheshti University of Medical Sciences, Tehran, Iran; 2 Public Health Department, Local Health Unit N.2 “Marca Trevigiana”, 31100 Treviso, Italy; 3 Human Genetics Research Center, Baqiyatallah University of Medical Sciences, Tehran, Iran; 4 Hematology and Oncology Research Center, Tabriz University of Medical Sciences, Tabriz, Iran; 5 JC School of Public Health and Primary Care, Chinese University of Hong Kong, Hong Kong, China; 6 Department of Pediatrics, Iran University of Medical Sciences, Tehran, Iran; 7 Department of Clinical Pathology, Faculty of Veterinary Medicine, University of Tehran, Tehran, Iran; 8 Department of Parasite Vaccine Research and Production, Razi Vaccine and Serum Research Institute, Agriculture Research, Education and Extension Organization (AREEO), Karaj, Iran; 9 Health Research Center, Baqiyatallah University of Medical Sciences, Tehran, Iran; 10 Nephrology and Urology Research Center, Baqiyatallah University of Medical Sciences, Tehran, Iran; 11 Abadan School of Medical Sciences, Abadan, Iran; 12 Department of Neurosurgery, University Medical Center, Tüebingen, Germany

**Keywords:** Gastrointestinal cancer, COVID-19, Angiotensin convertase enzyme 2, transmembrane serine protease 2, Pathophysiology

## Abstract

Recent studies on the pathophysiology of COVID-19 are indicating that the Angiotensin convertase enzyme 2 (ACE-2) and transmembrane serine protease 2 (TMPRSS2) can act as a major component in the fusion of SARS-Cov-2 with target cells. It has also been observed that the expression of ACE-2 and TMPRSS2 can be altered in malignancies. Shedding light on this matter could be crucial since the COVID-19 pandemic interfered with many gastrointestinal cancer screening programs. Herein we discuss the possibility of severe forms of COVID-19 in patients with gastrointestinal cancers due to the gastrointestinal entry route of SARS-CoV-2 into the human body. The disruption of cancer screening programs caused by the current COVID-19 pandemic could therefore have massive negative health impact on patients affected by gastrointestinal malignancies.

## Background

In 2020, the COVID-19 pandemic challenged the healthcare systems of more than 200 countries, resulting in the death of more than 1.5 million people worldwide [1]. In several countries, the exponential surges in the number of COVID-19 cases during the second and third wave of the pandemic resulted in healthcare systems reaching their capacity [2]. The shortage of medical supplies and healthcare resources, occupation of hospital wards and increased risk of nosocomial COVID-19 infections interfered with many medical procedures such as elective surgeries, screening for cancer and even vaccinations [3]. Albeit several commercial COVID-19 vaccines  are currently available, achieving herd immunity through vaccination is still a debated endpoint. With that in mind, several vaccination guidelines have been developed to prioritize sub-groups vulnerable to COVID-19 [4, 5]. Arranging these guidelines require deep knowledge of the COVID-19 pathophysiology, which is continuously evolving.

Whilst initial studies focused on respiratory symptoms of COVID-19, latest evidence suggested that gastrointestinal (GI) symptoms are usual and SARS-CoV-2 can be detected in feces in about 50% of infected subjects, although no clear association between GI symptoms and SASR-CoV-2 positivity of feces exists yet [6]. The most common GI symptoms include nausea, anorexia, diarrhea and vomiting and are associated with severe forms of the disease, extended hospitalizations and higher mortality risk [6–9]. Furthermore, it has been observed that SARS-CoV-2 could be isolated from stool samples even after testing negative for COVID-19 in the upper respiratory tract [10]. Several mechanisms have been proposed to explain the underlying causes of the GI symptoms associated with COVID-19, including disturbance of GI flora, drugs side effects and most importantly direct infection of enterocytes [11]. Novel evidence in fact suggests that SARS-CoV-2 can invade the enterocytes, where it can replicate and spread through fecal-oral transmission route [12, 13]. Understanding the underlying mechanism of direct invasion of the GI tract by SARS-CoV-2 could therefore help predict the conditions potentially influencing the clinical pattern of COVID-19.

In the early days of the pandemic, the imperfect risk assessment of COVID-19 postponed several preventative schemes as GI cancers screening programs due to the unknown nature and behavior of SARS-CoV-2, which initially was believed to be capable of invading only the lung parenchyma.

Herein, we discuss how malignant transformation can make patients with GI tumors more susceptible to direct invasion by SARS-CoV-2 and more vulnerable to severe forms of COVID-19, thus questioning the rationale of discontinuing cancer screening programs, especially against GI cancers.

## Discussion

### The role of ACE-2 and TMPRSS2 in SARS-CoV-2 cellular invasion

**Fig. 1:**
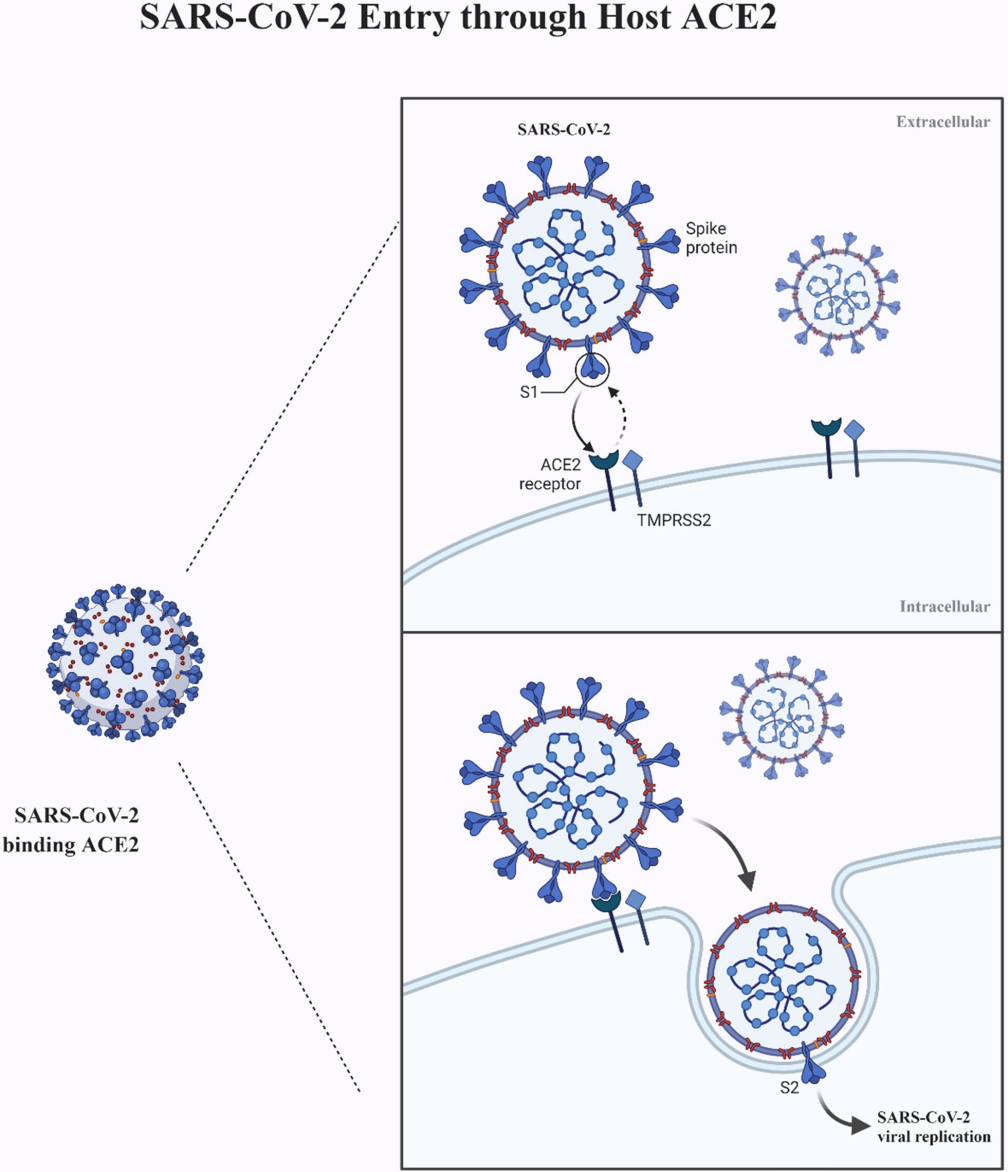
The infection mechanism of SARS-CoV-2 through ACE-2 cell receptor

Investigating the pathophysiology of COVID-19 and cellular pathways contributing to viral invasion, proliferation and immunogenicity not only supports the search for a cure against the disease but also helps stratifying the morbidity and mortality risk for COVID-19, defining susceptible sub-groups. One of the initial leading hypotheses of COVID-9 transmission was that SARS-CoV-2 invades target cells via the Angiotensin Converting Enzyme-2 (ACE-2) receptor [14] (Fig. 1).

ACE-2 is a single-pass type 1 membrane-bound enzyme of several epithelial lineage cells of various tissues, including the lung, GI tract, liver, kidney and brain. The latter X-linked enzyme, which is bound to a Zinc ion, consists of an extracellular enzymatic domain, a transmembrane chain and an intracellular C-terminal portion [15]. ACE-2, an important part of the renin-angiotensin system, is not only known for its role in regulating blood pressure by hydrolyzing and turning Angiotensin 2 into Angiotensin [1–7], but it also contributes to many physiological processes such as inflammation, tissue development and neuro-degeneration [16]. In the renin-angiotensin system ACE-2 downregulates ACE. Whilst Angiotensin 2 binds with AT1 receptor to induce vasoconstriction, inflammation and fibrosis, Angiotensin [1–7] acts on Mas receptors (MasR) inducing powerful vasodilation and anti-proliferative as well as anti-apoptotic action [17].

**Fig. 2:**
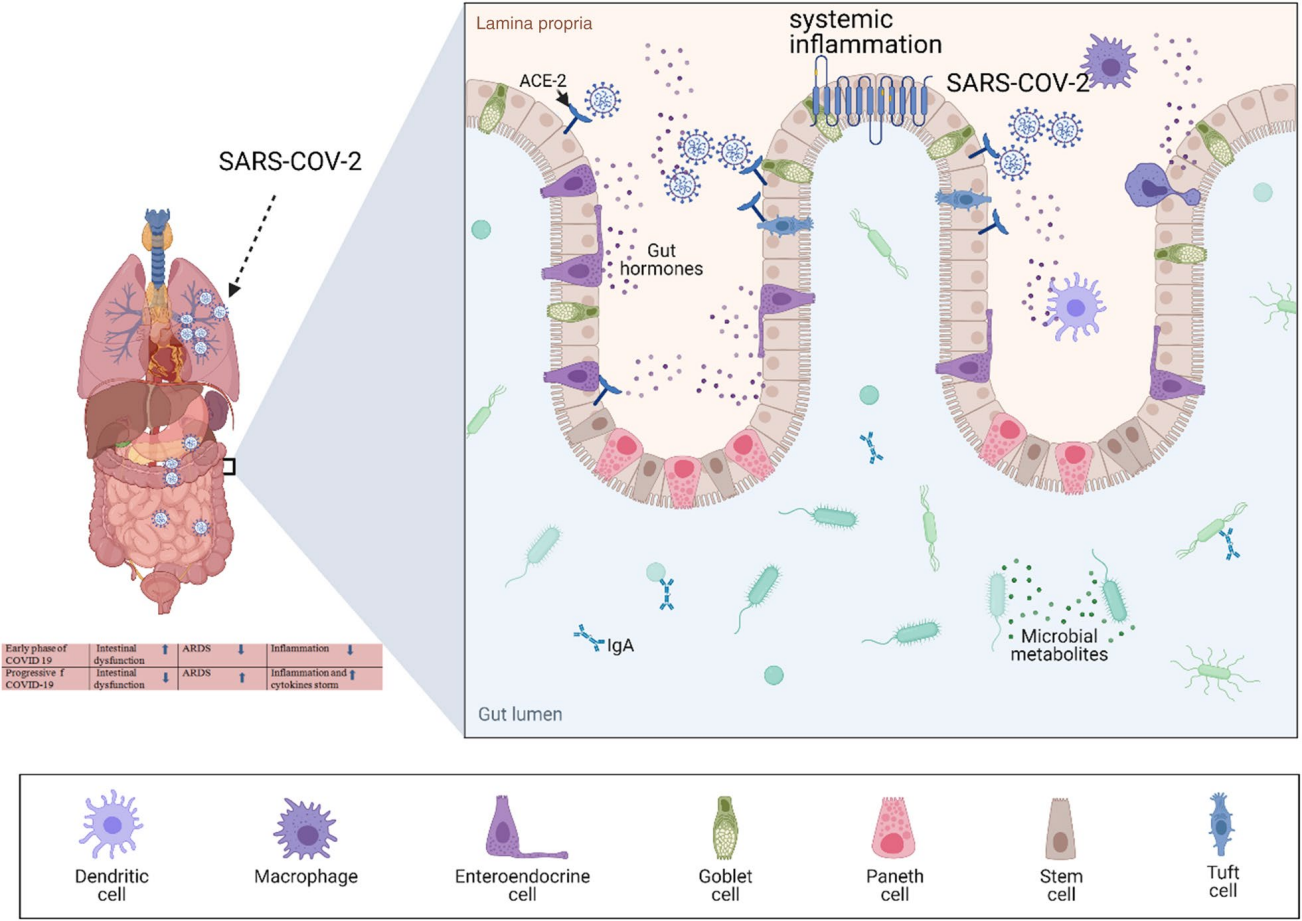
SARS-CoV-2 uses ACE-2 receptor to enter the absorptive enterocytes of the ileum and colon, causing the typical gastrointestinal symptoms of COVID-19

ACE-2 cell receptor was confirmed as the port of entry for SARS-CoV-1 into target cells [14–16, 18]. Since SARS-CoV-1 and SARS-CoV-2 share a high degree of homology, ACE-2 was immediately indicted as the main potential entry route also of SARS-CoV-2 infection of target cells from the early stages of the COVID-19 pandemic [19]. As shown in Fig. 2, SARS-CoV-2 binds with target cells through its Spike (S) protein, which is cleaved and primed by the host transmembrane serine protease 2 (TMPRSS2) and the disintegrin and metalloproteinase 17 ADAM17 [20, 21]. In addition to Spike proteins of SARS-CoV-1 and SARS-CoV-2, TMPRSS2 cleaves and activates also influenza virus hemagglutinin [22–24]. Co-expression of ACE-2 and TMPRSS2 could be the GI entry route for SARS-CoV-2 not only in pneumocytes but also in absorptive enterocytes of ileum and colon and may account for the frequent GI symptoms of COVID-19 [6, 25].

Severe forms of COVID-19 are seemingly triggered by an immunological imbalance leading to severe acute respiratory syndrome (SARS) and cytokine storm [26]. ACE-2 receptor  has  a vital function in the pathogenesis of COVID-19, activating the coagulation system (causing thrombophilia), the renin-angiotensin system (leading to cardiovascular instability) and the kinine–kallikrein system (causing acute inflammatory lung oedema) [27, 28]. Several manifestations of COVID-19, such as cardiovascular, kidney and brain symptoms, are associated with expression of ACE-2 and TMPRSS2 [29–31]. With that in mind, finding populations and conditions with upregulated expression of ACE-2 could be useful to detect susceptible sub-groups for targeted prevention of severe forms of COVID-19.

Since elevated expressions of ACE-2 and TMPRSS2 are associated with higher mortality risk from COVID-19, the latter two receptors have been investigated to develop potential agents effective to interfere with the replication of SARS-CoV-2 [32–34]. Targeting TMPRSS2 by a protease inhibitor could in fact be an anti-COVID-19 strategy blocking SARS-CoV-2 cell infection [18, 35].

### Expression of ACE-2 and TMPRSS2 in malignant transformation

**Fig. 3:**
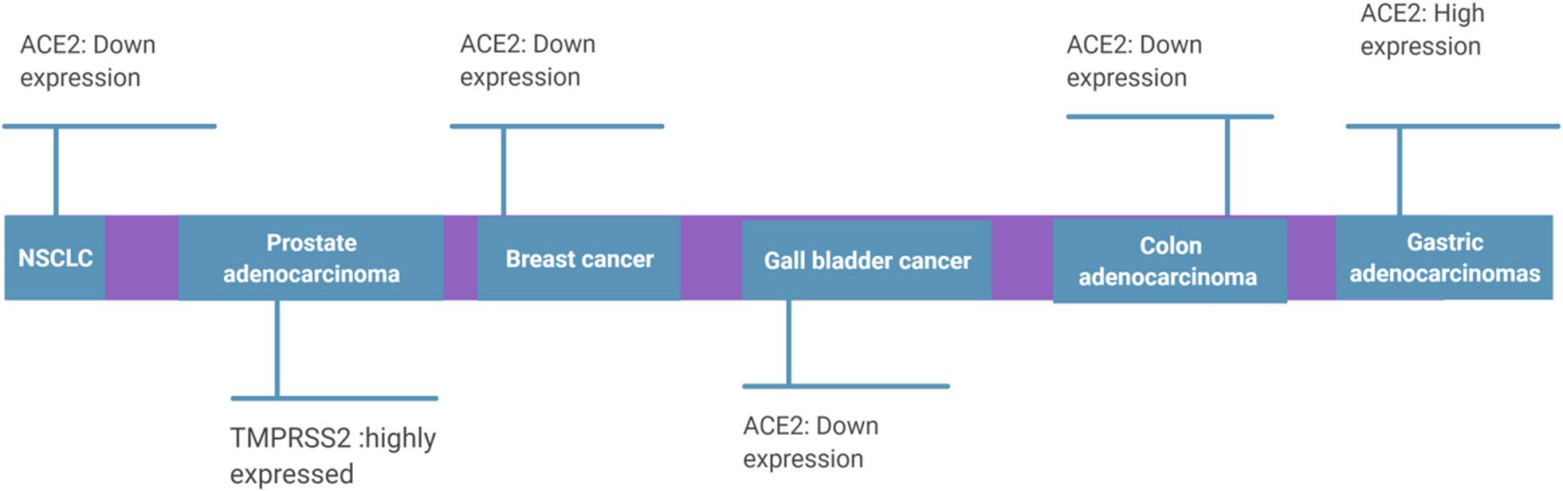
Expression of ACE2 and TMPRSS2 in tumor cells can be quite different from normal tissue cells

Expression of ACE-2 and TMPRSS2 in tumor cells may be remarkably different from normal tissue cells (Fig. 3).

ACE2 is highly expressed in various cancers including, squamous cell/adenosquamous carcinoma, endometrial carcinoma, colorectal, breast, prostate and lung cancer [36–38] as well as prostate adenocarcinoma [39].

Expression of ACE-2 and  health outcome can be quite variable in different solid malignancies, depending on the stage and the underlying origin of the tumor [40]. The analysis of the Cancer Genome Atlas (TCGA) and public data revealed ACE-2 over-expression in many kinds of malignancies, including rectum adenocarcinoma, cervical cancer, pancreatic adenocarcinoma, kidney renal papillary cell carcinoma and kidney renal clear cell carcinoma, when compared with adjacent tissues, while ACE-2 downregulation was observed in liver, breast and prostate cancers [41]. By using the Oncomine and Tumor Immune Estimation Resource (TIMER) databases, PrognoScan, GEPIA and Kaplan-Meier plotter databases, ACE-2 over-expression was found to be associated with higher overall survival in uterine corpus endometrial carcinoma (Hazard Ratio = 0.47; 95%CI: 0.30–0.73; Logrank p = 0.0007), kidney renal papillary cell carcinoma (Hazard Ratio = 0.44; 95%CI: 0.24–0.81; Logrank p = 0.0063), lung adenocarcinoma (Hazard Ratio = 0.60; 95%CI: 0.44–0.82; Logrank p = 0.0011) and liver hepatocellular carcinoma (Hazard Ratio = 0.55; 95%CI: 0.38–0.80; Logrank p = 0.0017) [42]. The same study also reported a positive correlation between ACE-2 expression and level of immune infiltration by B cells (R^2^ = 0.166; p < 0.010), CD4+ T cells (R^2^ = 0.154; p < 0.010), neutrophils (R^2^ = 0.223; p < 0.001) and dendritic cells (R^2^ = 0.271; p < 0.001) in uterine corpus endometrial carcinoma. For kidney renal papillary cell carcinoma ACE-2 expression was positively correlated with degree of macrophage infiltration (R^2^ = 0.322, p < 0.001) [42]. Adjusting for tumor stage, presence of metastases, pathological state and histological grade, over-expression of ACE-2 was notably correlate with  longer overall survival (Hazard Ratio = 0.8259; 95%CI: 0.7734–0.8819; Logrank p < 0.0001) and relapse-free survival (Hazard Ratio = 0.8023; 95%CI: 0.7375–0.8729; Logrank p < 0.0001) in clear cell renal cell carcinoma patients, using TCGA, GEO and TIP database [43].

The expression of ACE-2 in cancerous cells has been subject to speculations, although ACE-2 upregulation seems to have a protective role against tumour progression being  associated with favorable prognosis [37, 38]. Whilst AT1 receptor promotes the angiogenesis by inducing VEGF production and uncontrolled cell proliferation of tumoral cells [44], the interaction of Angiotensin [1–7] with MasR inhibits genesis, abnormal proliferation and progression of tumors [45, 46].

Since it could disrupt the production of vascular endothelial growth factor (VEGF), interfering with angiogenesis and tumor growth, ACE-2 upregulation hindered migration and invasion of breast cancer cells both in vivo and in vitro [47]. Negative ACE-2 expression was in fact associated with poor prognosis at multivariable Cox analysis on 46 patients affected by squamous cell/adenosquamous carcinoma of the gallbladder and 80 patients with adenocarcinoma of the gallbladder [48].

The activity of RAS oncogene, which influences cell proliferation and tumor growth, was upregulated in 19 patients affected by extrahepatic cholangiocarcinoma, resulting in significantly higher mean serum levels of ACE (56.6 ± 27.4 U/l) as compared to patients with choledocolithiasis (32.9 ± 14.6 U/l) and controls (28.6 ± 10.6 U/l) [49]. ACE inhibitors have been studied as potential cancer treatments. Captopril administered daily to mice via intraperitoneal injection (750 mg/kg; at a volume of 0.3 ml) dramatically decreased the number and size of liver metastases from colorectal cancer [50, 51]. Likewise, high daily doses of ACE inhibitors significantly reduced the risk of overall esophageal cancer (adenocarcinoma as well as squamous-cell cancer of the esophagus) by 45% (OR = 0.55; 95% CI: 0.33–0.93) in a population-based case-control study nested within the General Practitioners’ Research Database on individuals aged 40–84 years old from the UK during 1994–2001 [52].

Similar to ACE-2, TMPRSS2 is over-expressed in prostate adenocarcinoma, lung [53] and colorectal cancer, becoming an established tumour biomarker [37, 39, 53].

According to the Human Protein Atlas, ACE 2 and TMPRSS2 are highly expressed in prostate cancer and on tumor as well as normal colorectal epithelial tissues [36]. By contrast renal, urothelial, pancreatic and lung cancers showed low to moderate membranous or granular cytoplasmic immunoreactivity to the latter two proteins and their negative expression level was observed in other malignancies [48].

TMPRSS2 is capable of increasing the metastatic spread of prostate cancer by activating the Hepatocyte Growth Factor (HGF) [54]. TMPRSS2–ERG fusion in prostate tumors could be implicated in the activation of NOTCH pathway, capable of increasing proliferation and maintenance of progenitor cells. Chromatin immune-precipitation and parallel sequencing showed that TMPRSS2–ERG was involved in the development of prostate cancer through disruption of lineage-specific differentiation and potentiation of the EZH2-mediated de-differentiation program [55]. Ectropic expression of TMPRSS2–ERG fusion was found to be not only involved in alteration of chemo-sensitivity, but also in chemo-responsiveness to androgens in prostate cancer, depending on cell line and fusion type [56]. Binding and expression change of the latter gene via SARS-CoV-2 could therefore be pursued as cancer treatment [48].

Since increased expression of TMPRSS2 stimulates androgen-driven prostate cancer progression, therapeutic approaches directed toward inhibition of TMPRSS2 have been suggested to reduce the risk of metastatic progression in patients with prostate cancer [54]. The transcription of TMPRSS2 and ACE2 in major organs could be modulated by systemic androgen deprivation among adult male mice, prompting camostat mesylate and androgen regulation as a therapeutic strategy for cancer patients infected by SARS-CoV-2 [57].

Among 9280 patients with confirmed SARS-CoV-2 infection from 68 hospitals of the Veneto Region (North-Eastern Italy), those affected by prostate cancer and not receiving androgen-deprivation therapy were significantly more susceptible to COVID-19 (OR = 4.05; 95%CI: 1.55–10.59) than those receiving androgen deprivation (which decreases TMPRSS2 expression) [58].

### Expression of ACE-2 in gastrointestinal malignancies

Over-expression of ACE-2 and TMPRSS2 in tumor tissues may render them more vulnerable to SARS-CoV-2 infection [42, 54]. According to data from TCGA and Genotype-Tissue Expression (GTE), ACE2 and TMPRSS2 were found to be potentially implicated in the genetic susceptibility to SARS-COV-2 among cancer patients [59].

In GI organs, the interaction of SARS-CoV-2 with ACE-2 results in more damage to the mucous membrane barrier and subsequent inflammatory cytokine response [60]. It has been suggested that the ACE-2 receptor could even be used by SARS-CoV-2 to enter cholangiocytes, inflicting direct damage to these cells [61].

The COVID-19 pandemic requires special attention on patients affected by GI tumors—malignancies more common in the elderly—who already have higher ACE-2 and TMPRSS2 expression and are more likely to be affected by  other comorbidities that make them more susceptible to severe forms of COVID-19 [62]. There is evidence that ACE-2 expression upregulated in a wide variety of adenocarcinomas, including GI tract’s carcinoma. Bernardi et al. observed that MasR and ACE-2 expression/activity were both upregulated in colon adenocarcinoma cells as compared to controls (p < 0.001) or non-neoplastic colon mucosa resected 5 cm from tumour borders (p < 0.005) [63].

It is also argued that the expression of ACE-2 increases with malignancy grade, being higher in adenocarcinoma than adenoma of the colon. Growing expression level of ACE2 has been reported from healthy individuals to patients affected by adenoma or colorectal cancer, who were more likely to be infected by SARS-CoV-2 than the former at analysis of ACE-2 RNA expression in a cohort study and other databases, implying intestinal tropism of SARS-CoV-2 [64].

This progressive upregulation was also observed for the stomach, where ACE-2 expression increases from gastritis to metaplasia and gastric adenocarcinomas [60].

As already mentioned, a higher risk of SARS-CoV-2 infection involves cancer patients, where a gradual increase of ACE-2 expression was found at Bulk tissue RNA sequencing and single-cell RNA sequencing of public data [60].

Among 52 pre/asymptomatic COVID-19 patients affected also by GI cancer at hospital admission—median age of 62.5 years—higher expression of ACE-2 was confirmed by immunofluorescence as compared with the general population. ACE2 was found to be remarkably expressed in enterocytes or macrophages of the appendix, rectum and colon, and the mortality rate was reported to be higher in COVID-19 patients (16.7%) than COVID-19 free patients' [65]. Higher expression of ACE-2 may therefore influence the risk of GI tract tumors. Different types of cytokines are produced by T helper cells against infectious diseases [66]. Long-term chronic conditions may contribute to malignant changes of the GI mucosa mediated by cytokines, and severe COVID-19 is frequently associated with cytokine storm syndrome [60].

The exact underlying mechanism for the upregulation of ACE-2 in GI adenoma and carcinoma is still unknown and several potential explanatory mechanisms have been suggested, including local inflammation in metaplasia [60], since expression of ACE-2 and TMPRSS2 were found to be enhanced in the rectum of patients with inflammatory bowel disease [67]. Nevertheless, ACE2 and TMPRSS2 expression sustained by inflammatory bowel disease is seemingly region-specific across the entire intestine, being found to be reduced in the inflamed ileum [67].

Dysbiosis has been linked with several diseases [68–70], including GI cancers, local inflammation due to malignant transformations, and chemotherapy [71–73]. Recent studies have suggested that dysbiosis can increase the mortality risk of COVID-19 patients, by altering the ACE-2 pathway [74]. It has been described that the complex of ACE-2 combined with sodium-dependent neutral amino acid transporter (B(0)AT1) can influence the regulation of GI flora [74]. In addition, an *in vivo* study by Costa et al., revealed USF1 gene expression as a new central regulator of DNA damage against *helicobacter pylori* (Hp) infection and this gene is associated with patient prognosis in gastric cancer [75].

### ACE-2 and TMPRSS2 polymorphisms

TMPRSS2 is expressed in Type II alveolar cells, alveolar macrophages and bronchial epithelial cells, whereas it is not expressed by Type I alveolar cells [76]. Single nucleotide polymorphisms (SNPs) are involved in over-expression of TMPRSS2 [77]. Since the lung is considered one of the primary target locations of SARS-CoV-2, it is argued that TMPRSS2 expression levels in pulmonary cells change across different populations, with consequent variable susceptibility to COVID-19.

In a German case control study on 239 positive and 253 negative SARS-CoV-2 patients recruited from 11 March-31 October 2020, TMPRSS2 rs383510 variant was significantly associated with enhanced risk of SARS-CoV-2 infection (OR = 2.00; 95%CI: 1.30–3.08; p = 0.002). In a second multivariable model, male sex was the sole independent predictor [OR = 2.64; 95%CI 1.44–4.84; p = 0.002) of COVID-19 severity [78]. Nevertheless, the role of TMPRSS2 gene polymorphism on SARS-CoV-2 risk requires further confirmatory evidence on larger studies and different populations [78].

ACE-2 polymorphisms have been associated with several co-morbidities, including malignancies, essential hypertension (G8790A polymorphism) and cerebrovascular accidents in patients affected by type 2 diabetes mellitus. Exomics analysis in native and mixed South American populations and silico genomics databank assessment of other populations revealed extensive ACE2 polymorphisms, which could have an impact on clinical manifestations and outcomes of COVID-19 [79]. In particular, ACE-2 polymorphism could be linked to multi-organ failures in COVID-19 patients [80] and may cause mild to severe forms of the disease in certain groups [81], influencing the respective  prevalence and mortality rate [82]. The log-transformed prevalence (R^2^ = 0.410; p < 0.0001) and mortality (R^2^ = 0.457; p < 0.0001) for COVID-19 in 33 countries (on April 1, 2020) negatively correlated with ACE-1 D allele frequency, taking into account the start of the epidemic in each country [83].

ACE-2 I/D polymorphism, which has been found to be associated with comorbidities such as diabetes and hypertension, is suspected to be a causal factor in severe forms of COVID-19 [84, 85]. In a genetic study on 64 Egyptian patients, 40 with hypertension and 24 with type 2 diabetes, the D allele of ACE gene was associated with increased risk of hypertension and/or diabetes than DI allele (OR = 3.00; 95%Cl 0.993–9.067) and II allele (OR = 4.250; 95%CI 1.234–14.630a) than healthy controls [85]. Conversely, the D allele was associated with  increased  risk of hypertension (OR = 3.13; 95%Cl :1.405–6.978) and diabetes (OR = 4.14; 95% CI: 1.615–10.622) than the I allele [85].

Analyzing 349 worldwide population samples from Allele Frequency Database (ALFRED), ID and DD ACE polymorphisms were found to be associated with enhanced ACE/Ang-II activity, increased blood pressure and severe acute respiratory distress syndrome among COVID-19 patients as compared with other genotypes [86] As already mentioned, ACE-2 polymorphism can be more common in some GI cancers and can also promote malignant mutations. For instance, in a Chinese study contrasting 241 colorectal cancer patients with 299 non-cancer controls enrolled from April 2008 to October 2010, those carrying the ACE D allele were more likely to develop undifferentiated tumors (OR = 1.54; 95%CI: 1.04–2.28) and metastasis (OR = 1.56; 95%CI: 1.08–2.26) as compared to those carrying the ACE I genotype, although there was no remarkable correlation among cases and controls [87].

In a study comparing 113 gastric cancer patients (24 with the ACE II allele and 32 with the DD genotye) and 189 controls with no gastric cancer, a significantly higher number of lymph node metastases (p < 0.001) and higher Unio Internationale Contra Cancrum (UICC) tumor stage (p = 0.01) were associated with the DD genotype as compared with the II allele. Furthermore, ACE was expressed by endothelial cells in 100% collected specimens of gastric cancer at immunohistochemistry [88].

Although no association between H. pylori positivity or stomach atrophy and ACE polymorphism was found in a Japanese study on 202 gastric cancer patients and 454 healthy controls, the risk of gastric cancer was significantly higher  among patients with I/D genotype affected by atrophic gastritis caused by Helicobacter pylori (OR = 1.59; 95%CI: 1.02–2.48) [89]. A German study compared the genomic DNA from 88 patients with early gastric cancer confined either to the mucosa or submucosa with 145 non-cancer controls. II ACE genotype featured by low activity (OR = 0.20; 95%CI: 0.08–0.54; p = 0.009) and ID/II with intermediate/low activity (OR = 0.55; 95%CI: 0.31–0.96; p = 0.044) were significantly less expressed than the reference (DD allele with high activity) [90].

Combining all the above evidence, ACE I/D polymorphism may play different roles, depending on the type of cancer. There is a possibility that specific ACE-2 polymorphisms, more common in patients with GI cancers, may make these patients more susceptible  to severe forms of COVID-19 and related adverse outcomes such as multi-organ failure. Furthermore, some similarities between signaling cellular pathways in GI malignant transformations and COVID-19 may arguably exist.

### Suspension of gastrointestinal cancer screening programs in COVID-19 pandemic

In several countries, a sequential scheme for early diagnosis of colorectal cancer in adults older than 55 years has been deployed [91]. The COVID-19 pandemic interfered with many screening and prevention programs for GI cancers [92]. In March of 2020, the American Cancer Society suspended all cancer screening programs on average-risk individuals until further update, due to rising number of COVID-19 cases [93]. The latter restrictions were extended to colonoscopy, which is the prevalent screening approach against colorectal cancer in the USA [93]. In the UK the reduction of endoscopy procedures performed during the COVID-19 pandemic ranged from 84% in Wales to 88% in England as compared to before the pandemic [94]. Alternative strategies are being pursued to postponing the management of cancer patients and rearrange tumour treatment strategies [95]. Although the number of screening colonoscopies performed in Italy during lock down (March 9–May 4) significantly decreased compared to year 2019, in a retrospective observational study the detection rate of colorectal cancer (p = 0.002) and high-risk adenomas (p-0.001) by screening colonoscopies was significantly higher during the pandemic. This may suggest that the COVID-19 pandemic resulted in unnecessary health care delays and missed GI cancer cases [96]. It has been estimated that the cancer diagnostic delays due to the COVID-19 pandemic can result in a 15.3–16.6% increase in mortality rates for colorectal in UK [97]. There is moderate evidence that a 30 + days delayed resection of colorectal cancer is associated with lower survival [98]. With that in mind, screening programs should not completely cease during the COVID-19 pandemic, but rather continuing in COVID-19-free healthcare facilities.

## Conclusions

Although COVID-19 already had an extensive and immediate impact on burdened healthcare systems, its long-term effect on mortality and morbidity of the respective patients are yet to be elucidated. As discussed above, patients with GI cancers can be more vulnerable to COVID-19 than the general population due to higher expression of ACE-2 and TMPRSS2, which can serve as entry route for SARS-CoV-2 into target cells. On one hand, postponing screening procedures can be considered a way of protecting the vulnerable groups against COVID-19, by limiting their exposures to SARS-CoV-2 in healthcare facilities. Nonetheless, postponing cancer screening programs can enhance the morbidity and mortality risk attributable to GI malignancies, rendering these patients also more susceptible to COVID-19, since ACE-2 expression in enterocytes progressively increases with malignancy stage.

Cancer patients are considered one of the most vulnerable populations for COVID-19. It is therefore essential to maintain tailored screening programs against GI cancers during the current pandemic.

Although several of the current COVID-19 vaccines did not include cancer patients in their clinical trials and data on side effects of these vaccines in cancer patients are still unavailable, it is recommended to prioritize these patients in the COVID-19 vaccination programs.

## Data Availability

Not applicable.
